# Inhibition of Immunoproteasome Attenuates NLRP3 Inflammasome Response by Regulating E3 Ubiquitin Ligase TRIM31

**DOI:** 10.3390/cells13080675

**Published:** 2024-04-13

**Authors:** Yubin Lee, Boran Yoon, Sumin Son, Eunbin Cho, Kyung Bo Kim, Eun Young Choi, Dong-Eun Kim

**Affiliations:** 1 Department of Bioscience and Biotechnology, Konkuk University, 120 Neungdong-ro, Gwangjin-gu, Seoul 05029, Republic of Korea; dldbqls6834@naver.com (Y.L.); boranyoon0519@gmail.com (B.Y.); smson119@naver.com (S.S.); 7bini@naver.com (E.C.); 2 Department of Cellular & Molecular Medicine, Herbert Wertheim College of Medicine, Center for Translational Science at Port St. Lucie, Florida International University, 11350 SW Village Pkwy, Port St. Lucie, FL 34987, USA; kykim@fiu.edu

**Keywords:** inflammatory bowel disease, NLRP3 inflammasome, immunoproteasome inhibitor, tripartite motif-containing protein 31

## Abstract

Excessive secretion of pro-inflammatory cytokines leads to the disruption of intestinal barrier in inflammatory bowel disease (IBD). The inflammatory cytokine tumor necrosis factor alpha (TNFα) induces the assembly of the NLRP3 inflammasome, resulting in the augmented secretion of inflammatory cytokines implicated in the pathogenesis of inflammatory bowel disease (IBD). TNFα has also been known to induce the formation of immunoproteasome (IP), which incorporates immunosubunits LMP2, LMP7, and MECL-1. Inhibition of IP activity using the IP subunit LMP2-specific inhibitor YU102, a peptide epoxyketone, decreased the protein levels of NLRP3 and increased the K48-linked polyubiquitination levels of NLRP3 in TNFα-stimulated intestinal epithelial cells. We observed that inhibition of IP activity caused an increase in the protein level of the ubiquitin E3 ligase, tripartite motif-containing protein 31 (TRIM31). TRIM31 facilitated K48-linked polyubiquitination and proteasomal degradation of NLRP3 with an enhanced interaction between NLRP3 and TRIM31 in intestinal epithelial cells. In addition, IP inhibition using YU102 ameliorated the symptoms of colitis in the model mice inflicted with dextran sodium sulfate (DSS). Administration of YU102 in the DSS-treated colitis model mice caused suppression of the NLRP3 protein levels and accompanied inflammatory cytokine release in the intestinal epithelium. Taken together, we demonstrated that inhibiting IP under inflammatory conditions induces E3 ligase TRIM31-mediated NLRP3 degradation, leading to attenuation of the NLRP3 inflammatory response that triggers disruption of intestinal barrier.

## 1. Introduction

Inflammatory bowel disease (IBD), a persistent inflammatory disorder affecting the gastrointestinal (GI) tract, arises from multifaceted interactions among genetic, environmental, and immunological factors. This interaction leads to dysregulated mucosal immune responses, resulting in excessive inflammation and tissue damage [1,2]. These factors compromise the protective functions of the intestinal epithelium [3]. The epithelium of the GI tract comprises tight junction barriers formed by individual cells, which effectively prevent the passage of harmful microorganisms and gastric acid [4,5]. Disruption of these barriers by pathogens or a spectrum of stress factors leads to tissue damage and increased intestinal permeability, ultimately contributing to the initiation of IBD [6,7].

Several studies have demonstrated the pivotal role of the inflammatory cytokine such as tumor necrosis factor alpha (TNFα) in mediating key pro-inflammatory pathways across inflammatory and autoimmune disorders including IBD [8,9,10]. TNFα initiates the nuclear factor-kappa B (NF-κB) signaling pathway, which results in the upregulation of the inflammasome sensors, such as the NOD-like receptor family pyrin domain containing 3 (NLRP3), at both transcriptional and translational levels [11,12]. The NLRP3 protein, in conjunction with apoptosis-associated speck-like protein containing a CARD (ASC) and pro-casepase-1, collaboratively constitute an active NLRP3 inflammasome [13,14]. Upon activation, caspase-1 facilitates the production of pro-inflammatory cytokines, interleukin-1β (IL-1β) and IL-18, ultimately resulting in their secretion and initiation of inflammatory signaling cascades [15,16]. Excessive release of IL-1β leads to elevated permeability of the intestinal barrier, as indicated by increased IL-1β levels observed in individuals diagnosed with IBD [17,18].

TNFα stimulation induces activation of the immunoproteasome (IP) as well as the NLRP3 inflammasome [19,20]. IP contains three catalytic subunits that are distinct from the constitutive proteasome: low molecular mass polypeptide-2 (LMP2), low molecular mass polypeptide-7 (LMP7), and multicatalytic endopeptidase complex subunit-1 (MECL-1) [21]. Since IP is a critical regulator of inflammatory responses, inhibitors targeting IP have emerged as prospective anti-inflammatory treatments [22,23,24]. In our previous study, inhibition of IP activity with the LMP2-selective inhibitor YU102, a peptide epoxyketone molecule, effectively reduced NLRP3 inflammasome formation, contributing to the mitigation of disrupted cellular junctions in inflammatory conditions within intestinal epithelial cells [25]. However, the specific molecular mechanism of how IP inhibition attenuates NLRP3 inflammasome formation remains to be elucidated. Therefore, we aimed to investigate the molecular basis of the interplay between IP and NLRP3 inflammasome formation using the LMP2-specific inhibitor YU102.

In the TNFα-stimulated intestinal epithelial cells, we observed a decrease in NLRP3 protein levels upon IP inhibition. NLRP3 has been known to undergo K48-linked polyubiquitination for proteasomal or autophagic degradation. The E3 ubiquitin ligase tripartite motif-containing protein 31 (TRIM31) promotes K48-linked polyubiquitination and facilitates the degradation of NLRP3 by the proteasome [26,27,28]. In this study, we observed that IP inhibition increased TRIM31 protein levels, K48-linked polyubiquitination of NLRP3, and the interaction between NLRP3 and TRIM31. The reduction in NLRP3 protein levels by the IP inhibition attenuated the NLRP3 inflammatory pathway and subsequent disruption of cellular junctions. In the intestinal epithelium of the dextran sodium sulfate (DSS)-induced colitis mice, inhibition of IP activity significantly ameliorated disruptive symptoms by downregulating protein levels of NLRP3 and secretion of inflammatory cytokines. Thus, our study on IP inhibition regulating TRIM31-mediated NLRP3 degradation in intestinal epithelial cells provides valuable insights into potential therapeutic strategies for IBD.

## 2. Materials and Methods

### 2.1. Reagents and Antibodies

DSS was obtained from MP Biomedicals (Solon, OH, USA). Minimum Essential Medium (MEM, L0415), premium fetal bovine serum (FBS, S1480-500), and penicillin/streptomycin solution 100X (p/s, L0022) were obtained from Biowest (Riverside, MO, USA). Compound YU102, an LMP2-specific epoxyketone-based inhibitor, was prepared as described previously [29,30]. Recombinant Human TNF-alpha Protein (210-TA) was obtained from R&D Systems (Minneapolis, MN, USA). MG132 (M8699) was purchased from Sigma-Aldrich^®^ Solutions (Burlington, MA, USA). Opti-MEM^TM^/Reduced Serum Medium (31985070) was obtained by GIBCO (Waltham, MA, USA). TRIM31 siRNA (sc-76746) was obtained from Santa Cruz Biotechnology (Dallas, TX, USA). DAPI (4′,6-Diamidino-2-Phenylindole, Dihydrochloride, D1306), Lipofectamine^TM^ 3000 Transfection Reagent (L3000015), Alexa FluorTM555 goat anti-rabbit IgG (A21428), and Alexa FluorTM488 goat anti-mouse IgG (11001) were purchased from Invitrogen Life Technologies (Waltham, MA, USA). Recombinant Anti-GAPDH antibody (ab181602), Recombinant Anti-Vimentin antibody (ab92547), and VeriBlot for IP Detection Reagent (ab131366) were purchased from Abcam (Cambridge, MA, USA). NLRP3 Rabbit mAb (15101), K48-linkage Specific Polyubiquitin Rabbit mAb (8081), and E-cadherin Mouse mAb (14472) were obtained from Cell Signaling (Danvers, MA, USA). PSMB8 Antibody (PA1-972) and ZO-1 Antibody (40-2200) were obtained from ThermoFisher (Waltham, MA, USA). LMP2/PSMB9 Antibody (NB300-625), and NLRP3/NALP3 Antibody (NBP2-12446) were purchased from Novus Biologicals (Cambridge, UK). TRIM31 Polyclonal antibody (12543-1-AP) and NLRP3 Monoclonal antibody (68102-1) were purchased from Proteintech (Rosemont, IL, USA). Mouse anti-rabbit IgG-HRP (sc-2357), and ASC Antibody (sc-514414) were from Santa Cruz Biotechnology (Dallas, TX, USA).

### 2.2. Animals

Animal handling was authorized by the Institutional Animal Care and Use Committee (IACUC) at Konkuk University (approval number for this study, KU22255). The mice used were six-week-old male C57BL/6 mice obtained from Orient Bio Inc. (Seongnam, Republic of Korea). These mice were housed under standard laboratory conditions, with a temperature of 22 ± 1 °C and a 12:12-h light/dark cycle.

### 2.3. Establishment of DSS-Induced Colitis

Colitis was induced by the oral administration of DSS. After one week of acclimatization, 7-week-old mice were randomly assigned to three groups (*n* = 6 per group): a control group (received water as vehicle), a 3% DSS-treated group, and a 3% DSS-treated with YU102 (5 mg/kg) group. For the administration of YU102 to each mouse, PBS (phosphate-buffered saline) was used as the vehicle with a volume of 200 μL. Administration of YU102 started with DSS treatment via intraperitoneal injection every 3 days for a total of 3 injections. Body weight was recorded daily during DSS treatment.

### 2.4. Cell Culture and Transfection

The human epithelial colorectal adenocarcinoma cell line Caco-2 was from the American Type Culture Collection (ATCC, Manassas, VA, USA). Caco-2 cells were grown in a 5% CO_2_ incubator at 37 °C, with MEM containing FBS 20%, and penicillin/streptomycin (p/s) 1%. To knock down the expression of TRIM31, Caco-2 cells were transfected with TRIM31 siRNAs using Lipofectamine^TM^ 3000 transfection reagent. The cells were seeded (1.5 × 10^5^) in a 6-well plate or seeded (8 × 10^4^) in a 12-well plate. After 24 h of incubation, the cell medium was replaced with Opti-MEM. After 2 h, cells were transfected with 50 nM siRNA at 37 °C for 9 h. The culture medium was then replaced with MEM containing FBS 20% and p/s 1%.

### 2.5. Proteasome Activity Measurements

Cells and tissue samples from mice were disrupted using a Passive Lysis Buffer (Promega) to release their contents. Through the Bradford assay, the protein concentration was quantified. Proteasome activity assay buffer (20 mM Tris-HCl, 0.5 mM EDTA, pH 8.0) was mixed with equal amounts of protein. Ac-PAL-AMC (S-310, BostonBiochem, Cambridge, MA, USA) as LMP2 fluorogenic substrates were then added to the assay solution at a concentration of 100 μM. A SpectraMax M5 microplate reader (Molecular Devices, San Jose, CA, USA) was used to measure the fluorescence emitted by the liberated AMC, with measurements taken every 1 min for a period of 60 min. The emission wavelength was set to 460 nm, and the excitation wavelength was set to 360 nm.

### 2.6. Hematoxylin and Eosin Staining

Tissue samples from the colon were fixed in 4% paraformaldehyde at 4 °C for 24 h, followed by embedding in paraffin. The 8 μm thickness tissue sections were prepared for staining with hematoxylin and eosin (H&E). The assessment of the H&E-stained tissue sections was conducted using a fluorescence microscope Axiovert 200M (Carl Zeiss, Oberkochen, Germany).

### 2.7. Western Blot Analysis

RIPA buffer was used to lyse mouse colon segments and Caco-2 cell pellets. Through the Bradford assay, the protein concentration was quantified. Proteins were separated on SDS-PAGE and then transferred to membranes. A specific amount of protein was separated using gradient SDS polyacrylamide gel electrophoresis (SDS-PAGE) and then transferred to membrane. Immobilon-P PVDF membranes (IPVH00010, Burlington, MA, USA) were used. The membrane was blocked for 1 h at 25 °C in TBST buffer containing 3% bovine serum albumin (BSA). The primary antibody was incubated with the membrane, followed by a 2 h incubation with secondary antibodies at RT. After the membrane was washed with TBST buffer, it was reacted with HRP Substrate Peroxide Solution and HRP Substrate Luminol Reagent (WBKLS0500, Millipore, Billerica, MA, USA). G:BOX Chemi XL (Syngene, Frederick, MD, USA) detected the signals.

### 2.8. Enzyme-Linked Immunosorbent Assay (ELISA)

Cells were seeded in a 12-well plate and treated with TNFα alone or in combination with YU102 (1 µM) and MG132 (10 µM). Mouse colon tissues were obtained from the control, 3% DSS, and 3% DSS with YU102 (5 mg/kg) treatment groups. The inflammatory cytokine IL-1β secreted from Caco-2 cells and mouse colon tissues was evaluated by the ELISA kit (CSB-E08053h, CUSABIO, Houston, TX, USA). The supernatant of cells and the proteins extracted from mouse colon tissue samples were added to the assay plate, and the assay was carried out according to the manufacturer’s guidance. The absorbance was measured at 450 nm using a VICTOR X3 multilabel plate reader (PerkinElmer, Waltham, MA, USA).

### 2.9. Immunocytochemistry

The cells were cultured on a Microscope Cover Glass (22 × 40 mm) placed in a 12-well plate and incubated under various conditions. The cells were fixed with 4% paraformaldehyde at 4 °C for 12 h. The cells were permeabilized using Triton X-100. After 20 min, blocking was performed with 3% BSA for 1 h. After incubation with primary antibodies at 4 °C for 12 h, the cells were incubated with fluorescence-conjugated secondary antibodies and DAPI for 2 h at 25 °C in the dark. Cells were mounted with ProLong^TM^ Gold antifade reagent (P36934, Invitrogen) on a microscopic glass slide and monitored using a Super-Resolution Confocal Laser Scanning Microscope (Carl Zeiss, LSM 800).

### 2.10. Caspase-1 Activity

A 24-well plate was seeded with cells, which were then incubated under different conditions. Caspase-Glo^®^ 1 Inflammasome Assay kit (Promega, Madison, WI, USA) was used to measure caspase-1 activity. A substrate catalyzed by caspase-1, Z-WEHD-aminoluciferin, was added to the supernatant of the cells. After incubation for 1 h at 25 °C, a VICTOR X3 multilabel plate reader (PerkinElmer, Waltham, MA, USA) was used to measure the luminescence.

### 2.11. Co-Immunoprecipitation

A Dynabeads Co-Immunoprecipitation kit (14321D, Thermo Fisher Scientific, Waltham, MA, USA) was used to isolate NLRP3 proteins from Caco-2 cells treated under various conditions. After the Dynabeads were linked to the anti-NLRP3 antibody, immunoprecipitation was performed. The cells were exposed to NLRP3 antibody-coupled Dynabeads at 4 °C for 45 min. The beads were separated from the lysates using a magnet. HPH EB buffer (0.5 M NH_4_OH and 0.5 mM EDTA) was added to elute the captured proteins. After immunoprecipitated proteins were separated by SDS-PAGE, analysis was performed using TRIM31 antibody and K48-linkage Specific Polyubiquitin antibody.

### 2.12. FITC–Dextran Permeability Assay

Cells were cultured in a 12 mm Transwell with a 0.4 μm pore polycarbonate membrane insert until a monolayer was formed in MEM containing FBS 20% and p/s 1%. After drug administration, the upper chambers were replenished with a fresh mixture of OPTI-MEM and 500 μL of serum-free medium containing FITC–dextran (FDA-100MG, Sigma-Aldrich^®^). A VICTOR X3 multilabel plate reader was used to measure the fluorescence of the released FITC. The wavelengths for excitation and emission were measured at 485 nm and 535 nm, respectively.

### 2.13. Statistical Analysis

The mean ± standard deviation (S.D.) of the replicated data is presented. To assess statistically significant differences between two non-parametric groups, an unpaired t-test was used. One-way analysis of variance (ANOVA) was used to evaluate the differences between various groups. A *p*-value below 0.05 (with a 95% confidence level) was considered statistically significant.

## 3. Results

### 3.1. Immunoproteasome Inhibition Alleviates the Symptoms of DSS-Induced Colitis in Mice

Dextran sodium sulfate (DSS) is widely utilized as a chemical agent to provoke human ulcerative colitis-like conditions in mouse models. The specific process by which DSS-induced colitis occurs in mice remains unclear but likely involves multiple factors, including disruption of the intestinal epithelial barrier and spread of pro-inflammatory substances from the intestinal lining to the underlying tissue [31,32]. Furthermore, DSS significantly upregulates immunoproteasome subunits and induces damage to lysosomes, resulting in activation of the NLRP3 inflammasome [33,34].

**Figure 1:**
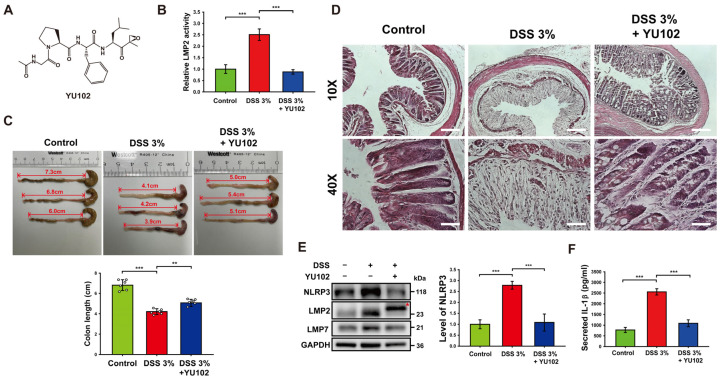
Treatment with YU102 mitigated the severity of DSS-induced colitis in mice by suppressing the NLRP3 inflammasome pathway. (**A**) Chemical structure of YU102, an LMP2-specific IP inhibitor. (**B**) The catalytic activity of LMP2 was measured in colon tissues from the control, 3% DSS-treated, and 3% DSS-treated with YU102 groups. *** *p* < 0.005. (**C**) Changes of colon length in mice. Shown are representative pictures of resected colons of mice. The bulging end of each colon is the mouse cecum. Colons were obtained from the control, 3% DSS-treated, and 3% DSS-treated YU102 groups (*n* = 6 per group). Colon lengths were measured from the bulging end of the cecum to the colon end, which are represented as double-arrowed line and depicted in the bar graphs. *** *p* < 0.005, ** *p* < 0.01. (**D**) Images of H&E staining of colon segments from the control, 3% DSS-treated, and 3% DSS-treated YU102 groups at 10× and 40× magnification. Scale bar: 10×, 200 μm; 40×, 50 μm. (**E**) Western blot analysis of NLRP3, LMP2, and LMP7 in colon tissues from the control, 3% DSS-treated, and 3% DSS-treated YU102 groups. The bar graph shows NLRP3 protein levels. *** *p* < 0.005. (**F**) Levels of IL-1β in the colon tissues from the control, 3% DSS-treated, and 3% DSS-treated YU102 groups were detected by ELISA. *** *p* < 0.005.

To investigate the effects of LMP2-specific inhibitor YU102 in murine models, 3% DSS was treated for 10 days, and YU102 was administered at 5 mg/kg via intraperitoneal injection every 3 days for a total of three injections. The specificity of IP inhibitor YU102 (Figure 1A) against LMP2 was assessed using a proteasome activity assay in colon segments with DSS-induced colitis (Figure 1B). The catalytic activity of LMP2 increased in the DSS-treated group compared to the control group but decreased in the DSS-treated group upon administration of YU102. Thus, YU102 specifically decreased activity of IP in the intestinal epithelium of the DSS-induced colitis model mice.

In the context of DSS-induced colitis, there is a well-established inverse relationship between colon shortening and severity of the condition [35]. To assess the potential beneficial effects of YU102 on colon length in DSS-induced colitis, we measured colon length in the control, 3% DSS-treated, and 3% DSS-treated with YU102 administration groups. As shown in Figure 1C, the length of colon was not relatively less decreased in the DSS-treated with YU102 administration group as compared to the DSS-treated group.

Next, we examined the effect of YU102 on histological changes in DSS-induced colitis colon segments. Figure 1D shows representative images of H&E staining of colon segments from the control, 3% DSS-treated, and 3% DSS-treated YU102 groups. In the colon segments of mice treated with 3% DSS, there was distortion in the crypt epithelium and an upsurge in the infiltration of acute and chronic inflammatory cells in the mucosa compared to the control group. However, the architectural structure of the crypts remained intact, and inflammatory reactions were significantly reduced in colon tissue segments from mice with DSS-induced colitis treated with YU102. Together, these observations indicate that YU102 prevents DSS-induced colitis symptoms such as shortening of colonic length. YU102 also ameliorates crypts epithelium distortion caused by DSS in colon tissues.

Numerous studies have found that NLRP3 inflammasome is a vital component of intestinal inflammation in a DSS colitis model [36,37]. To investigate whether YU102 could downregulate intestinal inflammation caused by DSS-induced activation of the NLRP3 inflammasome, we first examined the expression levels of NLRP3 and IP subunits in colon tissues. NLRP3 protein levels in colon tissues were observed to be increased in the DSS-treated group, whereas increased NLRP3 protein levels were not observed in the DSS-treated with YU102 administration group (Figure 1E). Levels of immunoproteasome subunits LMP2 and LMP7 were increased in the DSS-treated groups, irrespective of YU102 administration. Importantly, the LMP2 subunit band was observed to be shifted in the YU102-administered group (indicated with an asterisk in Figure 1E), reflecting that the molecular weight of LMP2 increased because of the covalent binding of YU102 to LMP2 subunit [30]. Thus, LMP2-specific inhibitor YU102 downregulates activation of the NLRP3 inflammasome by decreasing NLRP3 protein levels without altering immunoproteasome expression in the DSS-treated colitis model mice.

We next investigated whether immunoproteasome inhibition can decrease secretion of inflammatory cytokine IL-1β that is activated by the NLRP3 inflammasome. The levels of secreted IL-1β in colon tissues treated with DSS were significantly increased, while secretion of IL-1β was largely reduced when LMP2 inhibitor YU102 was administered in the DSS-treated mice (Figure 1F). These findings suggest that YU102 is effective in reducing the formation of the NLRP3 inflammasome and the subsequent generation of inflammatory cytokines in murine models induced by DSS.

### 3.2. Immunoproteasome Inhibitor Attenuates the NLRP3 Inflammasome Pathway in Intestinal Epithelial Cells

**Figure 2:**
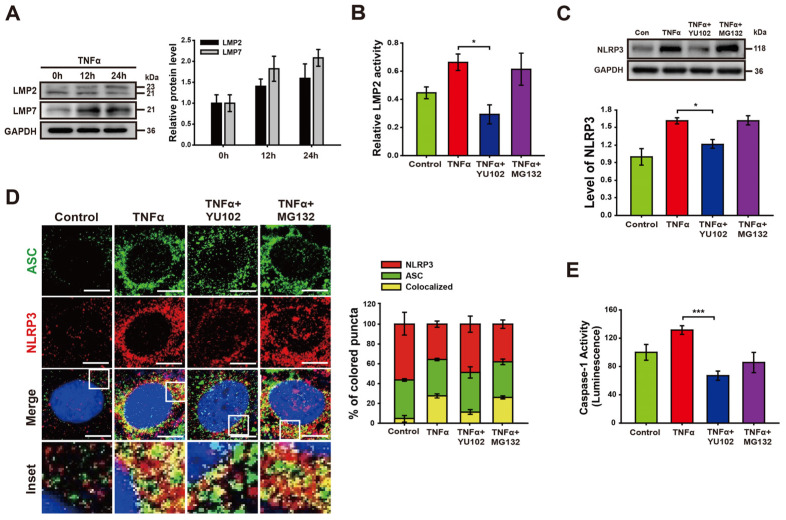
YU102 treatment attenuates NLRP3 inflammasome pathway in intestinal epithelial cells. (**A**) Western blot analysis of LMP2 and LMP7 in Caco-2 cells treated with 50 ng/mL TNFα for different times. The bar graph shows LMP2 and LMP7 protein levels. (**B**) LMP2-specific inhibition effect of YU102 in Caco-2 cells. Cells were treated with 50 ng/mL TNFα or 50 ng/mL TNFα in combination with 1 μM YU102 and 10 μM MG132 for 24 h. * *p* < 0.05. (**C**) Western blot analysis of NLRP3. The cells were incubated with 50 ng/mL TNFα or 50 ng/mL TNFα in combination with 1 μM YU102 and 10 μM MG132 for 24 h. The bar graph shows NLRP3 protein levels. * *p* < 0.05. (**D**) Confocal images depicting NLRP3 and ASC. The cells were treated with 50 ng/mL TNFα or 50 ng/mL TNFα in combination with 1 μM YU102 and 10 μM MG132 for 24 h. Scale bar, 10 μm. The bar graph shows the percentage of colored puncta in the merged images. (**E**) Caspase-1 activity in Caco-2 cells measured under various conditions. *** *p* < 0.005.

We investigated whether TNFα promotes activation of IP formation in Caco-2 cells (Figure 2A). The increasing exposure time to TNFα correlated with a gradual increase in the levels of LMP2 and LMP7, both integral subunits of the immunoproteasome. The specificity of the IP inhibitor YU102 against LMP2 was assessed in Caco-2 cell lysates (Figure 2B). Treatment with TNFα significantly augmented LMP2 activity, which was inhibited with IP inhibitor YU102. In contrast, the constitutive proteasome inhibitor MG132 did not effectively inhibit the activity of LMP2. Next, we examined the effect of the IP inhibitor on the NLRP3 protein levels in Caco-2 cells (Figure 2C). Co-treatment with TNFα and YU102 resulted in a decrease in NLRP3 protein levels compared to the TNFα-only treatment. In contrast to the significant decrease in NLRP3 levels observed with YU102 co-treatment, co-treatment with TNFα and MG132 did not affect NLRP3 protein levels. This indicates that the preferential inhibition of IP activity reduced the NLRP3 levels in the TNFα-treated inflammatory intestinal epithelial cells.

Next, we investigated whether IP inhibition prevents NLRP3 inflammasome assembly in intestinal epithelial cells (Figure 2D). NLRP3 inflammasomes were identified by merged fluorescence (yellow) resulting from the colocalization of NLRP3 (red) and ASC (green) fluorescences. Co-treatment with YU102 and TNFα resulted in a substantial reduction in yellow fluorescence compared to TNFα-only treatment. We additionally examined the catalytic activity of NLRP3 inflammasome in the presence of proteasome inhibitors (Figure 2E). TNFα treatment induced a significant increase in Caspase-1 activity, which was significantly decreased upon co-treatment with LMP2 inhibitor YU102 as compared to constitutive proteasome inhibitor MG132. These results suggest that IP inhibitor effectively suppressed TNFα-induced NLRP3 inflammasome assembly in Caco-2 cells.

### 3.3. Immunoproteasome Inhibition Increases K48-Linked Polyubiquitination of NLRP3

**Figure 3:**
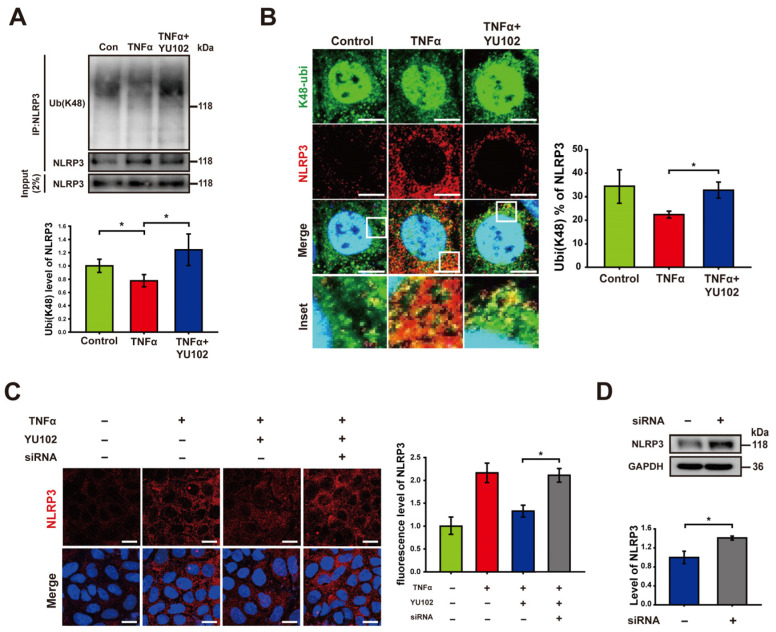
Immunoproteasome inhibition increases K48-linked polyubiquitination of NLRP3. (**A**) After co-immunoprecipitation with an NLRP3 antibody, immunoblot of NLRP3 and its K48-linked polyubiquitination obtained from cell lysates was presented. Cells were incubated with 50 ng/mL TNFα or 50 ng/mL TNFα in combination with 1 μM YU102 for 24 h. The bar graph shows the levels of co-immunoprecipitated K48-linked polyubiquitin with NLRP3. The intensity of each Ub (K48) band was normalized to that of NLRP3. * *p* < 0.05. (**B**) Confocal microscopy images of NLRP3 and K48-linked polyubiquitin in Caco-2 cells. Incubation with 50 ng/mL TNFα or 50 ng/mL TNFα in combination with 1 μM YU102 for 24 h. Scale bar, 10 μm. The bar graph shows colocalization puncta percentage of NLRP3 and K48-linked polyubiquitin to total puncta of NLRP3. * *p* < 0.05 (**C**) Confocal images depicting NLRP3 in intestinal epithelial cells. The cells were treated with 50 ng/mL TNFα or co-treated with 50 ng/mL TNFα and 1 μM YU102 for 24 h. Anti-TRIM31 siRNA (designated as ‘siRNA’)-transfected cells were treated with 50 ng/mL TNFα in combination with 1 μM YU102 for 24 h. Scale bar, 20 μm. The bar graph shows relative fluorescence levels of NLRP3. * *p* < 0.05 (**D**) Western blot analysis of NLRP3 expression in TRIM31 siRNA (designated as ‘siRNA’)-transfected cells treated with 50 ng/mL TNFα or 50 ng/mL TNFα in combination with 1 μM YU102. Bar graph shows NLRP3 protein levels. * *p* < 0.05.

Based on previous research indicating that NLRP3 undergoes polyubiquitination at the K48 residue, which leads to NLRP3 degradation by proteasomes or autophagy [38], we hypothesized that the reduction in NLRP3 observed following the IP inhibition is attributable to an increase in K48-linked polyubiquitination of NLRP3. To examine the plausible effect of the IP inhibitor on K48-linked polyubiquitination in Caco-2 cells, we employed co-immunoprecipitation to quantify the levels of K48-linked polyubiquitin that interacts with the NLRP3 (Figure 3A). K48-linked polyubiquitination of NLRP3 was increased in cells treated with YU102 compared to that in cells treated with TNFα alone. To further monitor K48-linked polyubiquitination of NLRP3, we monitored colocalization of NLRP3 and K48-linked polyubiquitin, in which NLRP3 labeled with red fluorescence and K48-linked polyubiquitin with green fluorescence were merged for the yellow fluorescence (Figure 3B). Consistent with the results from co-immunoprecipitation, we observed an increase in K48-linked polyubiquitination ratio of NLRP3 with low levels of NLRP3 protein in the YU102 treated condition compared to the TNFα-only treatment. This suggests that IP inhibition enhances the K48-linked polyubiquitination of NLRP3 with a significant decrease in NLRP3 protein levels in Caco-2 cells.

It has been reported that the E3 ubiquitin ligase TRIM31 directly interacts with NLRP3, facilitating K48-linked polyubiquitination and proteasomal degradation. TRIM31 suppression in a mouse model of IBD led to increased levels of NLRP3 [26,27,28]. We examined whether knockdown of TRIM31 could reverse the reduction in NLRP3 levels induced by IP inhibitor treatment (Figure 3C). The fluorescence levels of NLRP3 in cells exposed to TNFα and the IP inhibitor decreased in comparison to cells treated with TNFα alone. The reduced fluorescence of NLRP3 by co-treatment with TNFα and YU102 was significantly elevated by the TRIM31 knockdown using TRIM31-specific siRNA. Similar to this result, an increase in NLRP3 protein levels was also observed in the TRIM31 siRNA-transfected cells compared with that of untransfected cells (Figure 3D). Taken together, E3 ligase TRIM31 plays a role for K48-linked polyubiquitination of NLRP3, leading to a decrease in protein levels of NLRP3 in the TNFα-induced inflammatory Caco-2 cells treated with IP inhibitor YU102.

### 3.4. Inhibition of Immunoproteasome Activity Increases Protein Level of TRIM31

**Figure 4:**
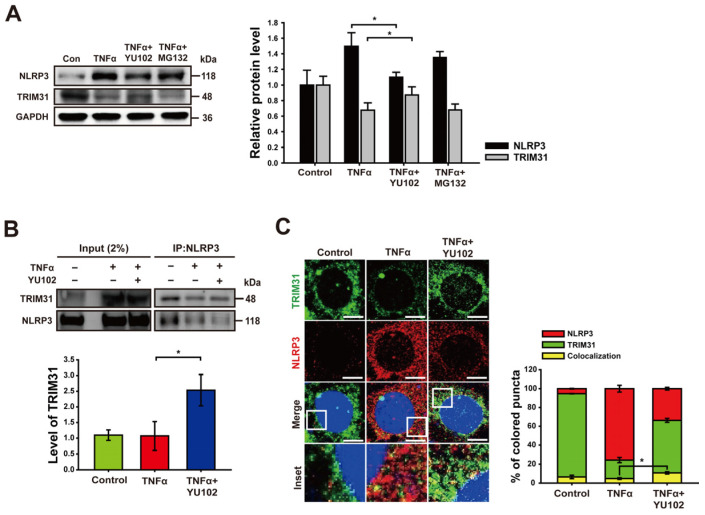
Inhibition of immunoproteasome upregulates the level of TRIM31. (**A**) Immunoblot analysis of NLRP3 and TRIM31 in intestinal epithelial cells. Treatment with 50 ng/mL TNFα or 50 ng/mL TNFα in combination with 1 μM YU102 and 10 μM MG132 for 24 h. The bar graph shows NLRP3 and TRIM31 protein levels. * *p* < 0.05. (**B**) After co-immunoprecipitation with NLRP3 antibody, immunoblot of NLRP3 and TRIM31 obtained from cell lysates was presented. Cells were incubated with 50 ng/mL TNFα or 50 ng/mL TNFα in combination with 1 μM YU102 for 24 h. Bar graph shows the levels of co-immunoprecipitated TRIM31 with NLRP3. The intensity of each TRIM31 band was normalized to that of NLRP3. * *p* < 0.05. (**C**) Confocal microscopy images of NLRP3 and TRIM31 in Caco-2 cells. A total of 50 ng/mL TNFα or 50 ng/mL TNFα in combination with 1 μM YU102 was treated for 24 h. Scale bar, 10 μm. Bar graph shows colocalization puncta of NLRP3 and TRIM31 to total puncta of NLRP3. * *p* < 0.05.

We next investigated whether IP inhibitor regulates the protein level of TRIM31 in inflammatory intestinal epithelial cells. Co-treatment with TNFα and YU102 resulted in a significant increase in TRIM31 protein levels compared to cells treated with TNFα alone (Figure 4A). In contrast, co-treatment with TNFα and MG132 indicated no change in TRIM31 levels compared to cells treated with TNFα alone. Conversely, the protein levels of NLRP3 decreased upon co-treatment with TNFα and YU102 compared to the TNFα treatment alone. This result suggests that IP inhibition leads to a decrease in NLRP3 protein levels by maintaining TRIM31 E3 ligase levels in the TNFα-induced inflammatory cells.

Next, we analyzed NLRP3 binding proteins to investigate whether IP inhibition not only increased TRIM31 protein levels, but also enhanced the interaction between NLRP3 and TRIM31. Co-treatment with TNFα and YU102 increased the interaction between NLRP3 and TRIM31 compared to that observed in the cells treated with TNFα alone (Figure 4B). Additionally, we labeled NLRP3 and TRIM31 with red and green fluorescence, respectively, enabling the tracking of their colocalization and individual expression levels. Compared to the cells treated with TNFα alone, in cells treated with TNFα and YU102 simultaneously, the fluorescence intensity of NLRP3 decreased, whereas the fluorescence intensity of TRIM31 increased. Furthermore, the interaction ratio between NLRP3 and TRIM31 was elevated in cells treated with both TNFα and YU102 compared to cells treated with TNFα alone (Figure 4C). Taken together, these results suggest that IP inhibition not only elevates TRIM31 protein levels but also enhances the interaction between TRIM31 and NLRP3, resulting in increased K48-linked polyubiquitination of NLRP3 and subsequent degradation.

### 3.5. Inhibition of Immunoproteasome Activity Alleviates Disruption of Intestinal Epithelial Barrier

**Figure 5:**
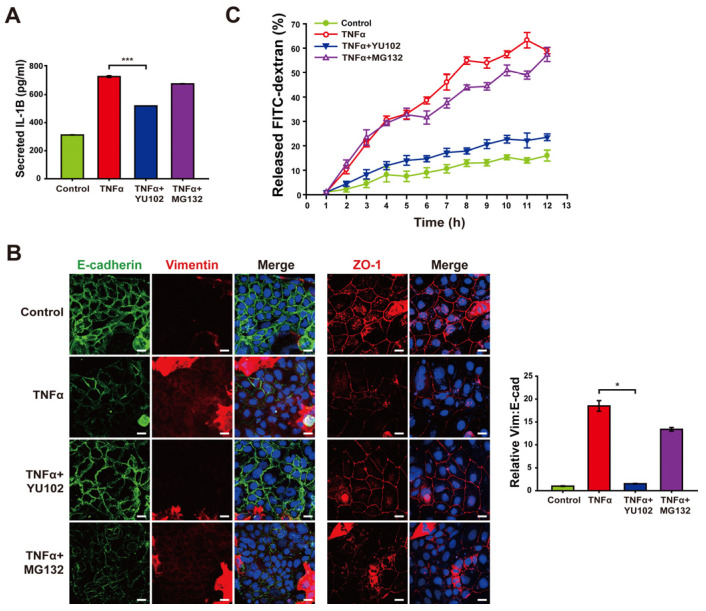
Treatment with YU102 mitigated the disruption of the intestinal epithelial barrier. Inhibition of immunoproteasome alleviates disruption of the intestinal epithelial barrier. (**A**) Secreted IL-1β levels in Caco-2 cells were measured using an ELISA kit. A total of 50 ng/mL TNFα or 50 ng/mL TNFα in combination with 1 μM YU102 and 10 μM MG132 were incubated with cells for 24 h. *** *p* < 0.005 (**B**) Confocal microscopic images of E-cadherin/Vimentin and ZO-1. Cells were incubated with 50 ng/mL TNFα or 50 ng/mL TNFα in combination with 1 μM YU102 and 10 μM MG132 for 24 h. Scale bar, 20 μm. The bar graph shows the relative ratios (Vim: E-cad). * *p* < 0.05. (**C**) FITC–dextran cell permeability assay. Treatment with 50 ng/mL TNFα or 50 ng/mL TNFα in combination with 1 μM YU102 and 10 μM MG132 for 24 h. After Caco-2 monolayers were treated with 100 μg/mL FITC–dextran, released FITC fluorescence was measured at regular interval of one hour.

Based on the mitigation of NLRP3 inflammasome activation by IP inhibitor, we investigated whether YU102 also downregulates the release of inflammatory cytokines from TNFα-induced inflammatory cells (Figure 5A). Elevated levels of secreted IL-1β were observed in the cells with TNFα treatment, whereas co-treatment with YU102 and TNFα resulted in a reduction of IL-1β secretion. This result indicates that IP inhibitor effectively suppressed TNFα-induced production of inflammatory cytokines via NLRP3 inflammasome activation in Caco-2 cells.

Next, we investigated whether YU102 also ameliorates disruption of intestinal epithelium through epithelial–mesenchymal transition (EMT) progression triggered by secreted cytokines in the Caco-2 cells. EMT has been known to be associated with the development of IBD, which contributes to the loss of cellular junctions and increased permeability of intestinal epithelial cells [39]. TNFα treatment resulted in the induction of EMT progression and loss of cellular tight junction, as evidenced by decreased E-cadherin expression with increased vimentin expression and loss of cellular tight junction protein ZO-1, respectively (Figure 5B). In contrast, co-treatment with TNFα and YU102 resulted in increased E-cadherin and decreased vimentin levels, suggesting that IP inhibition attenuates EMT progression induced by TNFα treatment. Furthermore, co-treatment with TNFα and YU102 prevented the TNFα-induced decrease in ZO-1 expression, maintaining cellular tight junctions.

To further substantiate the effect of YU102 on TNFα-induced disruption of the intestinal barrier, we utilized a FITC fluorophore-labeled dextran permeability assay to evaluate epithelial leakage (Figure 5C). In this assay, increased leakage in the intestinal epithelium may allow more FITC–dextran molecules to pass through the barrier, leading to higher fluorescence measured in the lower chamber. Co-treatment with TNFα and YU102 significantly reduced the release of FITC–dextran compared to TNFα-only treatment. In contrast, the proteasome inhibitor MG132 was ineffective in preventing disruption of the epithelial barrier, as evidenced by the substantial release of FITC–dextran. Hence, these results indicate the significant efficacy of IP-specific inhibitors in maintaining cellular junctions of intestinal epithelial cells by mitigating the NLRP3 inflammasome pathway that are prevalent in the intestinal epithelium inflicted with inflammatory cytokines.

## 4. Discussion and Conclusions

We observed a decrease in NLRP3 protein levels upon IP inhibition in the TNFα-stimulated intestinal epithelial cells. NLRP3, known to undergo K48-linked polyubiquitination for proteasomal or autophagic degradation [38,40,41,42], exhibited elevation of K48-linked polyubiquitination in cells treated with YU102. Next, we observed an elevation in E3 ubiquitin ligase TRIM31 upon the inhibition of the IP activity in the TNFα-stimulated intestinal epithelial cells. TRIM31 is involved in K48-linked polyubiquitination and subsequent degradation of NLRP3, a prominent component of the NLRP3 inflammasome. YU102, a peptide epoxyketone LMP2-specific inhibitor, decreased the protein levels of NLRP3 and increased the protein levels of TRIM31. Thus, YU102 treatment impeded the assembly of the NLRP3 inflammasome and suppressed Caspase-1 activity, thereby mitigating the release of pro-inflammatory cytokine IL-1β in TNFα-stimulated Caco-2 cells. In addition, IP inhibition ameliorates the progression of EMT and suppresses leakage in intestinal barrier permeability. These molecular details support our observation that inhibition of IP effectively mitigated the symptoms associated with DSS-induced colitis, including colonic length reduction and spleen enlargement in the IBD model mice. Analysis of colon tissues in the mice treated with DSS showed that inhibition of IP activity resulted in decreased NLRP3 protein levels with attenuated secretion of the pro-inflammatory cytokine IL-1β.

The pro-inflammatory cytokine TNFα induces both activation of the NLRP3 inflammatory pathway and conversion of constitutive proteasome to IP in intestinal epithelial cells [8,19]. The upregulation of NLRP3 transcription via NF-κB activation induced by TNFα is a well-established mechanism, contributing to the NLRP3 inflammasome pathway [43,44]. The NLRP3 inflammasome is a complex comprising three primary components: NLRP3, ASC, and Caspase-1. NLRP3, the core protein of the NLRP3 inflammasome, incorporates a central nucleotide-binding and oligomerization domain that is critical for both the assembly and ATPase activity. In addition, Caspase-1, the effector protein of the NLRP3 inflammasome, converts pro-IL-1β and pro-IL-18 into the active forms IL-1β and IL-18, respectively [45]. According to clinical studies, augmented inflammatory cytokine IL-1β secretion is associated with the severity of colonic tissues in IBD patients [46,47]. In addition, the absence of either Caspase-1 or NLRP3 in mice alleviated the severity of colitis, which correlated with diminished levels of IL-1β and IL-18 [36,48]. Hence, NLRP3 inflammasome contributes to the pathogenesis of IBD by mediating the secretion of inflammatory cytokines, including IL-1β and IL-18. IP inhibition has been investigated as a potential treatment agent for inflammatory disorders [49,50]. In the context of systemic lupus erythematosus, the efficacy of the linear epoxyketone peptide KZR-616, targeting both LMP7 and LMP2 subunits, has been assessed [51]. The effectiveness of YU102, a specific inhibitor of LMP2, in improving cognitive behavior in mouse models of Alzheimer’s disease and attenuating the secretion of inflammatory cytokines from microglial cells has been evaluated [30]. Additionally, in TNFα-stimulated intestinal epithelial cells, YU102 exhibited suppression of NLRP3 inflammasome formation [25]. Based on our observation that specific IP inhibition upregulates the protein level of E3 ligase TRIM31 that induces K48-linked polyubiquitination of NLRP3 with subsequent proteasomal degradation, it is very reasonable to suggest an IP-specific inhibitor as a potential reagent to preferentially attenuate NLRP3 inflammasome-mediated inflammation response. Because both constitutive proteasome and immunoproteasome are present in the cells that are stimulated with inflammatory cytokines, selective inhibition of IP is preferable to not compromise the constitutive proteasomal system. It is very likely that IP directly degrades TRIM31 under TNFα-induced inflammatory cells. Thus, it is tempting to investigate whether TRIM31 is a genuine substrate for IP under inflammatory conditions, which will be conducted as a further study. In addition, use of primary cells obtained from human colon epithelium is warranted in addition to Caco-2 cells, which are extensively employed for investigating intestinal epithelial cells. To expand our findings in the DSS-treated animal model, it would be worthwhile to test various colitis models, including TNBS, Oxazolone, or IL-10 knockout mice, to corroborate results observed in the DSS-induced colitis mice model.

**Figure 6:**
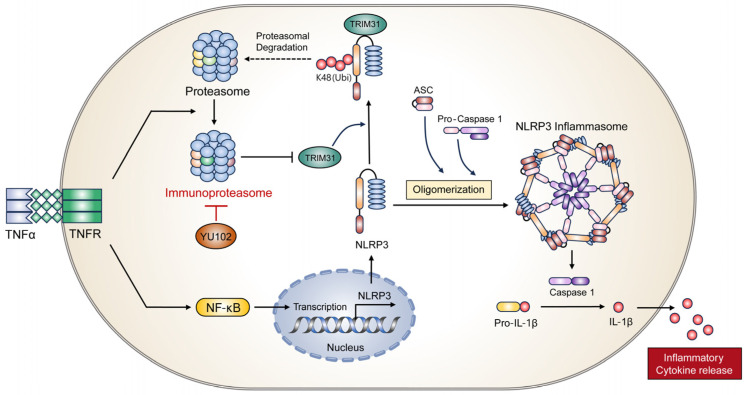
Proposed mechanism of action of IP inhibitor YU102 on NLRP3 inflammasome activation in TNFα-stimulated intestinal epithelial cells through TRIM31 regulation.

In conclusion, we elucidated a molecular mechanism underlying the interaction between IP and the NLRP3 inflammatory response using the LMP2-specific inhibitor YU102 (Figure 6). Specific IP inhibition upregulates protein level of E3 ligase TRIM31, which in turn promotes the interaction between NLRP3 and TRIM31. Interaction between NLRP3 and TRIM31 enhances K48-linked polyubiquitination of NLRP3 and leads to the degradation of NLRP3. In addition, we observed the maintenance of intestinal epithelial cell permeability due to the inhibition of the NLRP3 inflammatory response though IP inhibition. Therefore, we present a new regulatory mechanism between the IP and NLRP3 inflammatory pathways and suggest IP inhibitors as potential drugs to prevent the progression of IBD under inflammatory conditions.

## Data Availability

Data generated or analyzed during the study are included in this submitted article.
